# Loss of nuclear NOTCH1, but not its negative regulator NUMB, is an independent predictor of cervical malignancy

**DOI:** 10.18632/oncotarget.24828

**Published:** 2018-04-10

**Authors:** Elenaé Vázquez-Ulloa, Ana Clara Ramos-Cruz, Diddier Prada, Alejandro Avilés-Salas, Alma Delia Chávez-Blanco, Luis A. Herrera, Marcela Lizano, Adriana Contreras-Paredes

**Affiliations:** ^1^ Programa de Maestría y Doctorado en Ciencias Bioquímicas, Universidad Nacional Autónoma de México, Ciudad Universitaria, Mexico City, México; ^2^ Unidad de Investigación Biomédica en Cáncer, Instituto Nacional de Cancerología-Instituto de Investigaciones Biomédicas, Universidad Nacional Autónoma de México, Mexico City, México; ^3^ Departamento de Informática Biomédica, Facultad de Medicina, Universidad Nacional Autónoma de México, Mexico City, México; ^4^ Departamento de Patología Quirúrgica, Instituto Nacional de Cancerología, Mexico City, México; ^5^ Departamento de Medicina Genómica y Toxicología Ambiental, Instituto de Investigaciones Biomédicas, Universidad Nacional Autónoma de México, Mexico City, México

**Keywords:** cervical cancer, cervical intraepithelial neoplasia, NOTCH1, NUMB, immunostaining

## Abstract

The participation of NOTCH signaling in invasive cervical cancer (ICC) remains controversial since both tumor suppressive and oncogenic properties have been described. Additionally, the role of NUMB, a negative regulator of NOTCH, remains unclear in ICC. We aimed to investigate the role of NOTCH1 and NUMB expression and their localization in cervical intraepithelial neoplasia (CIN) and ICC samples. A total of 144 biopsies were obtained from the Instituto Nacional de Cancerología, México from 2004 to 2017, and were subjected to immunohistochemistry for NOTCH1 and NUMB. We found that nuclear NOTCH1 expression was more frequently found in CIN samples compared with ICC (77.55% vs. 15.79%, *p* = 0.001). NUMB was almost exclusively found in the nucleus of CIN samples (32.65% vs. 6.32%, *p* = 0.001). Cytoplasmic expression of NOTCH1 (44.21%) and NUMB (35.79%) was the most frequent localization in ICC. Multivariable-adjusted analysis showed that the loss of nuclear NOTCH1 expression was an independent predictor of malignancy (β = –3.428, 95% confidence interval [95% CI] = –5.127, –1.728, *p* = 0.001). In contrast, the association between cytoplasmic NUMB expression and cervical cancer was lost after adjusting for nuclear NOTCH1 expression (β = 2.074, 95% [CI] = –0.358, 4.506, *P* = 0.094). Additionally, patients with cytoplasmic NOTCH1 expression showed a borderline association with longer overall survival (OS) than those with nuclear NOTCH1 expression (*P* = 0.08). Our data suggest that the loss of nuclear NOTCH1 but not NUMB might be an independent predictor of malignancy in cervical cancer.

## INTRODUCTION

Invasive cervical cancer (ICC) is the fourth most common malignancy among women worldwide, with an estimated 528,000 new cases and 266,000 deaths in 2012 and with 87% of cervical cancer deaths occurring in less developed regions [1]. Among Mexican women, ICC is the second most common neoplasia, just after breast cancer [2]. Infection with human papillomaviruses (HPVs) has been well documented as the main etiological factor for ICC since the viral genome is present in practically all cervical cancer tumors [3]. To date, 13 HPV genotypes have been defined as carcinogenic or high-risk (HR) viral types for cervical cancer [4]. The HPV viral oncoproteins E5, E6 and E7 can disrupt several host signaling pathways; for example, E6 can deregulate p53 and PDZ proteins and enhance the activation of cellular pathways such as PI3K, Wnt and Notch [5].

The Notch pathway is a highly conserved signaling system that plays a key role in cell differentiation, survival and proliferation [6]. In the canonical Notch pathway, a transmembrane Notch receptor (NOTCH 1–4) interacts with Delta-Serrate-Lag-type (Dll1, Dll3, Dll4, Jagged1 or Jagged2) ligands. This interaction triggers the sequential proteolytic cleavage of the Notch receptor, releasing the intracellular domain (NICD), which translocates to the nucleus and activates the transcription of target genes, including *Hes1, Hes5*, *Hey1, Cyclin D1* and *Myc* [7–9].

The participation of Notch signaling in cervical cancer remains controversial since both tumor suppressive [10–12] and oncogenic properties [13, 14] have been described. Talora *et al.* (2002) showed a lack of NOTCH1 expression in ICC samples and in cervical cell lines [11]. In contrast, Zagouras *et al.* (1995) and Yousif *et al.* (2015) found an increase in NOTCH1 expression throughout cervical cancer progression [14, 15]. Moreover, Jagged-1 and Delta-1 ligands have been reported as overexpressed in ICC and in cervical adenocarcinoma [16].

Several reports have suggested that NUMB is a negative regulator of NOTCH1 signaling [7–9]. The interaction of NUMB with NOTCH1 may result in increased NOTCH1 ubiquitination [17]. NUMB may also act as a scaffold for the E3 ligases Itch and Suppressor of Deltex Su(Dx) [7, 18, 19] and cooperates with α-adaptin (part of the endocytic AP2 complex), thereby promoting NOTCH1 endocytosis [8, 20]. In breast cancer, NUMB has been defined as a tumor suppressor protein [21–23]; nevertheless, its role in ICC is not clear. Chen *et al.* (2009) reported NUMB overexpression in cervical malignant lesions compared with normal epithelia, suggesting a role for NUMB in cervical cancer progression [24]. However, the relationship between NOTCH1 and NUMB in ICC is not clear.

To understand the role of NOTCH1 and its negative regulator NUMB in cervical cancer, we investigated the expression and localization of NOTCH1 and NUMB in samples from 144 patients with cervical intraepithelial neoplasia (CIN) and ICC obtained from the Instituto Nacional de Cancerología-México from 2004 to 2017 using immunohistochemistry and determined their role as predictors of malignancy in ICC.

## RESULTS

### Characteristics of patients

We recruited cases with available paraffin-embedded samples from women diagnosed with CIN or ICC at the Instituto Nacional de Cancerologia, Mexico from April 2004 to January 2017. A total of 49 CIN and 95 ICC paraffin-embedded tissue samples and their clinical data were collected. The demographic and clinical characteristics of the patients are shown in Table 1. Most patients were older than 30 years old in both groups, corresponding to 69.39% in the CIN group and 97.89% in the ICC group (*P* = 0.001). Smoking (10.20% vs. 11.57%) and alcohol consumption (0.00% vs. 4.21%) were not different among groups. A higher frequency of obesity was observed in CIN patients than in ICC patients (57.70% vs. 26.58%, *P* = 0.01). Additionally, hormone contraception usage was more common in the CIN group (58.62%) than in the ICC group (32.50%, *P* = 0.01). The number of previous sexual partners and the type of HPV were not different among groups. HPV types 16 and 18 were the most common in both groups (CIN vs. ICC: 63.15% vs. 51.06% for type 16; and 15.80% vs. 14.90% for type 18).

**Table 1:** Demographic and clinical characteristics of patients (*n* = 144) with CIN and ICC treated at the Instituto Nacional de Cancerología-México from 2004 to 2017

Variable	CIN (*n* = 49)		ICC (*n* = 95)		*P*-value
	*n*	(%)	*n*	(%)	
Age					
<30	15	(30.61%)	2	(2.11%)	**<0.001**
≥30	34	(69.39%)	93	(97.89%)	
Smoking status^a^					
Smokers	5	(10.20%)	11	(11.57%)	1.00
Non-smokers	44	(89.80%)	84	(88.43%)	
Alcohol consumption^b^					
Positive	0	(0.00%)	4	(4.21%)	0.36
Negative	49	(100%)	91	(95.79%)	
Body mass index, kg/m^2^					
Normal (18.5–24.9)	4	(15.38%)	31	(39.24%)	**0.01**
Overweigth (25–29.9)	7	(26.92%)	27	(34.18%)	
Obesity (≥30)	15	(57.70%)	21	(26.58%)	
Unknown^c^	23		16		
Hormone contraception usage					
Yes	17	(58.62)	13	(32.50%)	
No	12	(41.38)	27	(67.50%)	0.05
Unknown^c^	20		55		
Number of previous sexual partners					
1	16	(38.10%)	22	(64.71%)	0.61
≥2	26	(61.90%)	12	(35.29%)	
Unknown^c^	7		61		
Human Papillomavirus (HPV)					
16	12	(63.15%)	48	(51.06%)	
18	3	(15.80%)	14	(14.90%)	0.49
Others^d^	4	(21.05%)	32	(34.04%)	
Unknown^c^	30		1		e

CIN: Cervical intraepithelial neoplasia. ICC: Invasive cervical cancer ^a^Smoking was defined as any tobacco consumption during their lifetime. ^b^Alcohol consumption was defined as any alcohol intake per week. ^c^Data not reported in the clinical files, and thus, this category was not included in the comparisons ^d^Other HPVs include types 6, 11, 31, 43, 42, 45 and 58. Bold: statistically significant.

### NOTCH1 and NUMB expression and localization in CIN and ICC

**Figure 1:**
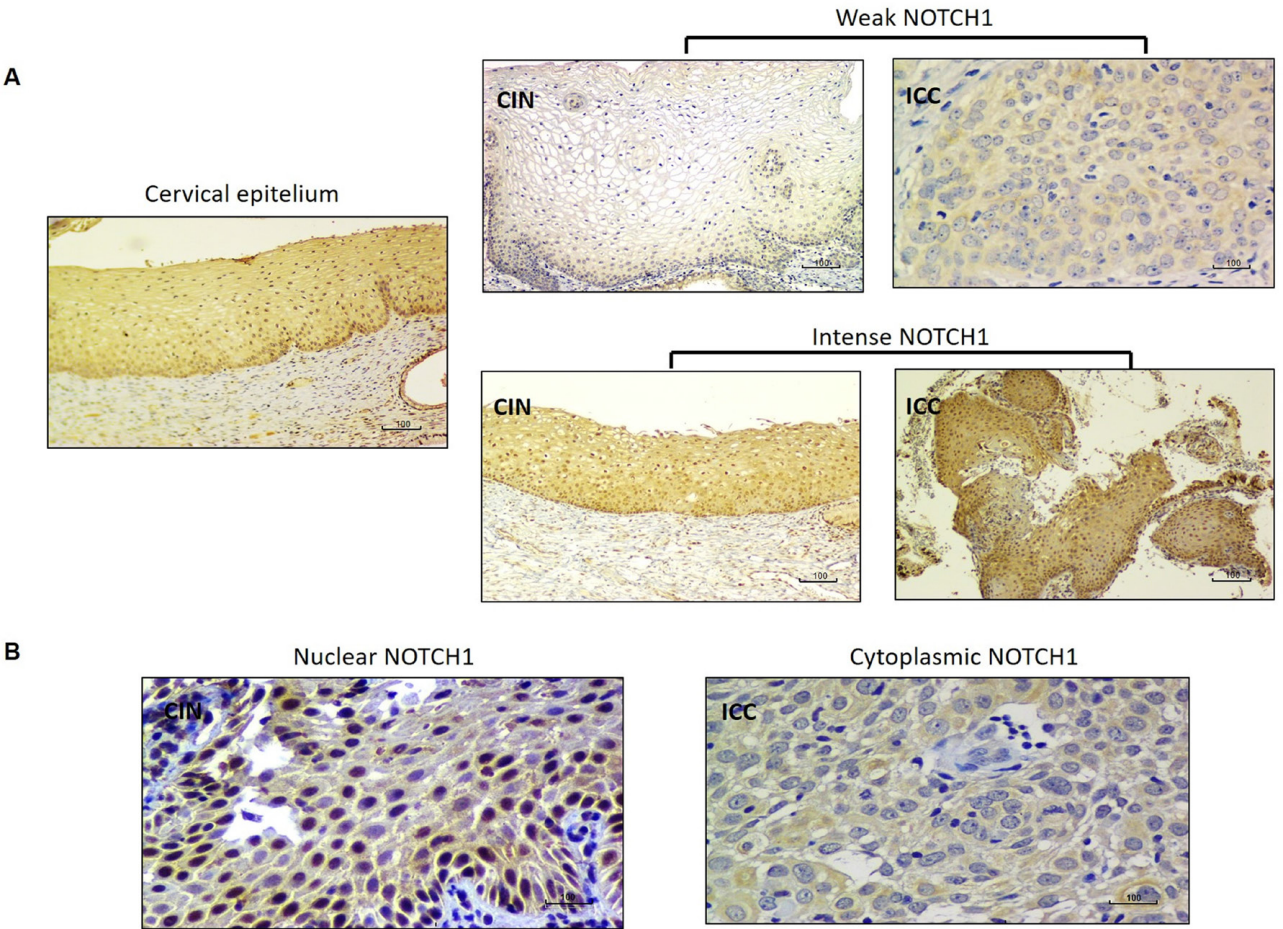
NOTCH1 immunostaining in CIN and ICC (**A**) Comparison of NOTCH1 immunostaining intensity (weak vs. intense) in CIN and ICC samples. (**B**) NOTCH1 immunostaining localization in the cell nucleus (CIN) and cytoplasm (ICC).

Normal cervical epithelium was used as positive control for NOTCH1 and NUMB expression in immunohistochemical analysis. Besides, confirmation of immunohistochemical results was done in a representative set of ICC samples with an alternative NOTCH1 antibody, obtaining 90% of concordance between antibodies, which confirms that the antibody used in this study is relievable (Supplementary Figure 1 and Supplementary Table 3). Accordingly to the protein Atlas database, the immunostaining of NOTCH1 in normal squamous cells is moderate with a homogenous distribution in the cell [25], similar to what we observed in normal epithelium (Figure 1A). Intense NOTCH1 expression was more frequently observed in CIN samples (22.45%) than in ICC samples (3.16%, *P =* 0.001) (Table 2 and Figure 1A). NOTCH1 nuclear staining was more frequently observed in CIN samples than in ICC samples (77.55% vs. 15.79%). Additionally, in ICC samples, NOTCH1 protein expression was mainly observed in the cytoplasm (44.21%), while no cytoplasmic case was observed in CIN (*P =* 0.001) (Table 2 and Figure 1B).

**Table 2:** NOTCH1 protein expression intensity and cellular localization in samples of patients (*n* = 144) with CIN and ICC treated at the Instituto Nacional de Cancerologia-Mexico from 2004 to 2017

	CIN (*n* = 49)		ICC (*n* = 95)		*P*-value
	*n*	(%)	*n*	(%)	
Intensity^a^					
Negative	10	(20.41%)	26	(27.37%)	
Weak	28	(57.14%)	66	(69.47%)	0.001
Intense	11	(22.45%)	3	(3.16%)	
Localization					
Negative	10	(20.41%)	26	(27.37%)	
Cytoplasm	0	(0.00%)	42	(44.21%)	˂0.001
Nucleus	38	(77.55%)	15	(15.79%)	
Cyto/nuc^b^	1	(2.04%)	12	(12.63%)	

CIN: Cervical intraepithelial neoplasia. ICC: Invasive cervical cancer. ^a^Intensity was categorized as weak = + and ++; and intense = +++ by immunohistochemistry. ^b^Presence of the protein in the cytoplasm and nucleus. Bold: statistically significant.

**Figure 2:**
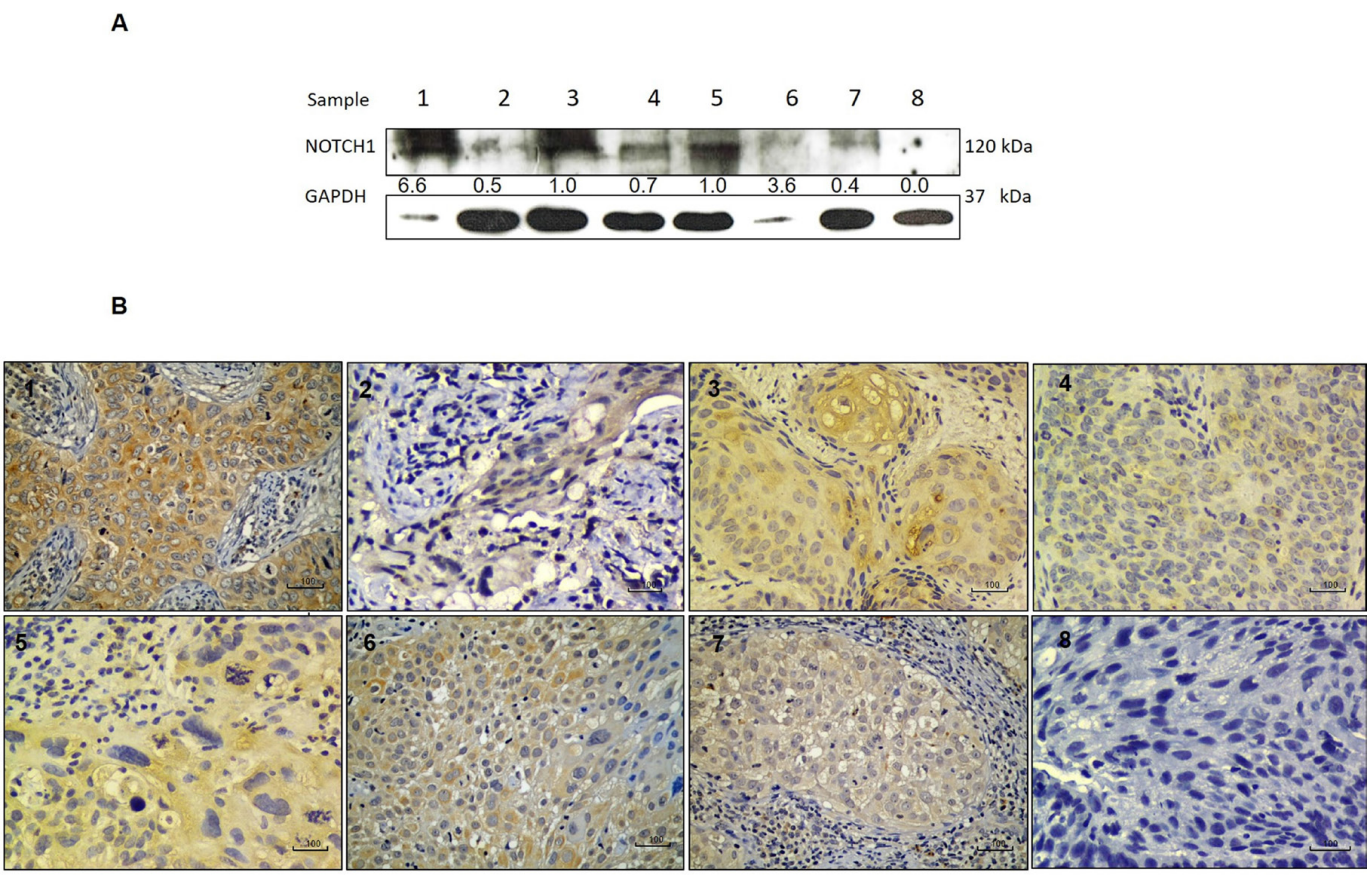
NOTCH1 protein expression and correlation with immunostaining in ICC (**A**) NOTCH1 protein expression analyzed by Western Blot in a set of cervical tumors showing intense (lanes 1, 6), moderate (lanes 3 and 5) and weak (2, 4 and 7) expression as well as negative expression (lane 8). (**B**) Immunostaining of corresponding samples showing intense (images 1 and 6), moderate (images 3 and 5), weak (images 2, 4 and 7) and negative (image 8) immunostaining of NOTCH1.

NOTCH1 protein expression was evaluated by Western Blot in eight representatives ICC cases (Figure 2A). The densitometric analysis showed that immunostaining intensity correlates with protein expression (Figure 2B). Relative NOTCH1 protein expression is higher in intense immunostained cases (samples 1 and 6), than in weak cases (samples 2, 3 and 4 and 7).

**Figure 3:**
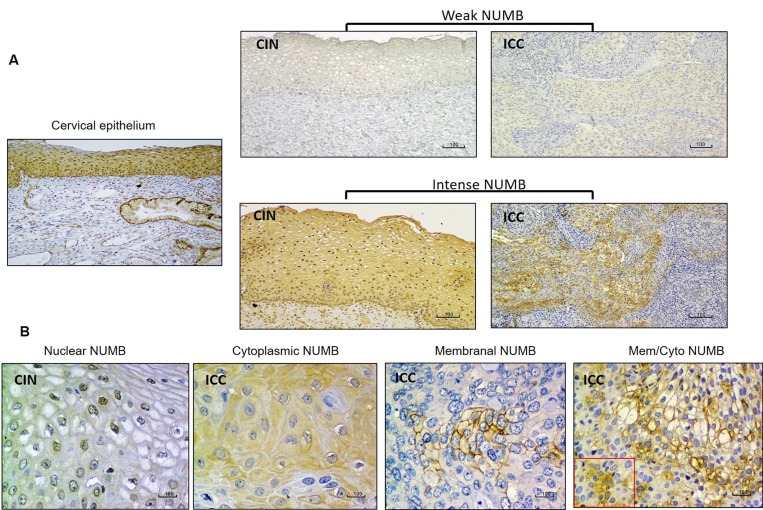
NUMB immunostaining in CIN and ICC (**A**) Comparison of NUMB immunostaining intensity (weak vs. intense) in CIN and ICC samples. (**B**) NUMB immunostaining localization in the cell nucleus (CIN); cytoplasm, membrane and membrane/cytoplasm (ICC).

For the NUMB protein, the normal epithelium showed weak cytoplasmic immunoreactivity (Figure 3A). Negative NUMB protein expression was more frequently observed in CIN samples than in ICC samples (65.31% vs. 42.11%, *P =* 0.014). Additionally, observations of the highest intensity for NUMB immunoreaction were more frequent in ICC than in CIN samples (0.00% in CIN vs. 6.31% in ICC) (Table 3 and Figure 3A). The NUMB protein, when present, was almost exclusively found in the nucleus in CIN samples (32.65% of nuclear NUMB in CIN vs. 2.04% of cytoplasmic NUMB), whereas in the ICC samples, its localization was heterogeneous, with a significant increase of NUMB expression in the cytoplasm (6.32% of nuclear NUMB in ICC vs. 35.79% of cytoplasmic NUMB) (Table 3 and Figure 3B).

**Table 3:** NUMB protein expression intensity and cellular localization in samples of patients (*n* = 144) with CIN and ICC treated at the Instituto Nacional de Cancerologia-México from 2004 to 2017

	CIN (*n* = 49)		ICC (*n* = 49)		*P*-value
	*n*	(%)	*n*	(%)	
Intensity^a^					
Negative	32	(65.31%)	40	(42.11%)	
Weak	17	(34.69%)	49	(51.58%)	0.014
Intense	0	(0.00%)	6	(6.31%)	
Localization					
Negative	32	(65.31%)	40	(42.11%)	
Membrane	0	(0.00%)	2	(2.10%)	
Cytoplasm	1	(2.04%)	34	(35.79%)	
Nucleus	16	(32.65%)	6	(6.32%)	˂0.001
Mem/cyto^b^	0	(0.00%)	8	(8.42%)	
Cyto/nuc^c^	0	(0.00%)	5	(5.26%)	

CIN: Cervical intraepithelial neoplasia. ICC: Invasive cervical cancer. ^a^Intensity was categorized as weak = + and ++; and intense = +++ by immunohistochemistry. ^b^Presence of the protein at the membrane and in the cytoplasm. ^c^Presence of the protein in the cytoplasm and nucleus. Bold: statistically significant.

### Relationship between NOTCH1 and NUMB expression

To determine if there was a relationship between the expression of NOTCH1 and NUMB (negative regulator of NOTCH1), we evaluated the expression, localization and positive tumoral area percentage of NOTCH1 and NUMB proteins in both CIN and ICC samples (Table 4). In CIN samples, the most frequent condition was NOTCH1-positive and NUMB-negative expression (44.90% vs. 26.32% for the same condition in ICC). In contrast, in ICC samples, the most frequent condition was NOTCH1-positive and NUMB-positive expression (34.69% in CIN vs. 46.32% in ICC). We also evaluated NOTCH1 and NUMB localization and we found that the most frequent combination was nuclear NOTCH1 and nuclear NUMB in CIN samples (75.51% vs. 16.84% in ICC). The most frequent condition observed in ICC samples was cytoplasmic NOTCH1 and nuclear NUMB (22.44% in CIN vs. 47.36% for the same condition in ICC). The distribution of frequencies for both expression and localization among the CIN and ICC groups were statistically significant (*P* = 0.014 for expression; and *P* = 0.001 for localization) (Table 4). Additionally, we evaluated the correlation between the distribution of NOTCH1 and NUMB throughout serial histological sections and found to be statistically significant in ICC but not in CIN (*r =* 0.226, *P =* 0.116 in CIN vs. *r =* 0.306, *P* = 0.002 in ICC) (Supplementary Figures 2 and 3).

**Table 4:** Demographic and clinical characteristics of patients (*n* = 144) with CIN and ICC treated at the Instituto Nacional de Cancerología-México from 2004 to 2017

Variable	CIN (*n* = 49)		ICC (*n* = 49)		*P*-value
	*n*	(%)	*n*	(%)	
Protein expression					
Negative^a^ NOTCH1 and negative^a^ NUMB	10	(20.41%)	15	(15.79%)	**0.014**
Negative^a^ NOTCH1 and positive^b^ NUMB	0	(0.00%)	11	(11.58%)	
Positive^b^ NOTCH1 and positive^b^ NUMB	17	(34.69%)	44	46.32%	
Positive^b^ NOTCH1 and negative^a^ NUMB	22	(44.90%)	25	(26.32%)	
Protein localization					
Nuclear^c^ NOTCH1 and nuclear^c^ NUMB	37	(75.51%)	16	(16.84%)	**0.001**
Nuclear^c^ NOTCH1 and cytoplasmic^d^ NUMB	1	(2.04%)	0	(0.00%)	
Cytoplasmic^d^ NOTCH1 and nuclear^c^ NUMB	11	(22.44%)	45	(47.36%)	
Cytoplasmic^d^ NOTCH1 and cytoplasmic^d^ NUMB	0	(0.00%)	34	(35.78%)	

CIN: Cervical intraepithelial neoplasia. ICC: Invasive cervical cancer. ^a^The negative condition was defined as an absence of immunostaining in the sample. ^b^The positive condition was defined as the presence of any immunostaining in the sample. ^c^Nuclear expression was defined as any positive nuclear immunostaining. ^d^Cytoplasmic expression was defined as any positive immunostaining outside of the cell nucleus. Bold: statistically significant.

### Association of NOTCH1 expression with malignancy status

Multivariable-adjusted analysis for the association between nuclear NOTCH1 expression and malignancy status showed a negative and significant association (β = –2.836, 95% CI = –3.694, –1.978, *P =* 0.001) (Table 5 and Supplementary Table 1). This association was consistent through sensitivity analyses, including age (β = –3.122, 95% CI = –4.180, –2,063, *P =* 0.001) and contraceptive consumption (β = –3.790, 95% CI = –5.459, –2.120, *P =* 0.001) as covariates (Supplementary Table 1). Moreover, the negative association of nuclear NOTCH1 with malignancy persisted even after adjustment for cytoplasmic NUMB expression (β = –3.428, 95% confidence interval [95% CI] = –5.127, 1.728, *P =* 0.001) (Table 5).

**Table 5:** Multivariable-adjusted model for the association between nuclear NOTCH1 expression and ICC diagnosis in patients with cervical cancer treated at the Instituto Nacional de Cancerologia-Mexico from 2004 to 2017 (*n* = 95)

Variable	β	(95% CI)	*P*-value	
NOTCH1 expression^a^	–3.428	(–5.27, –1.728)		**0.001**
Age	0.092	(0.015, 0.168)		**0.018**
HC	0.973	(**–**0.595, 2.541)		0.223
NUMB expression^b^	2.074	(**–**0.358, 4.506)		0.094

^a^Nuclear NOTCH1 expression was defined as any positive nuclear immunostaining at the cell nucleus. ^b^NUMB expression was defined as any positive immunostaining at the cell cytoplasm. Age was included as a continuous variable. HC: Hormonal contraception use. β = Estimate for the association between NOTCH1 expression and patient characteristics. 95% CI = 95% confidence interval. Bold: statistically significant.

### Association of NUMB expression with ICC

We also explored the association between NUMB expression and malignancy status through sensitivity analyses (Table 6 and Supplementary Table 2). In the univariable regression model, cytoplasmic NUMB expression was associated with cervical malignancy (β = 3.28, 95% CI = 5.310, 1.262, *P* = 0.001), and this association was persistent in the multivariable model adjusted by age (β = 3.487, 95% CI = 5.548, 1.427, *P =* 0.001) and contraceptive consumption (β = 2.946, 95% CI = 0.809, 5.082, *P* = 0.007) as covariates. Remarkably, when the model was adjusted by nuclear NOTCH1 expression, the significance of the association was lost (β = 2.074, 95% CI = –0.358, 4.506, *P =* 0.094) (Table 6).

**Table 6:** Multivariable-adjusted model for the association between cytoplasmic NUMB expression and malignancy in patients with cervical cancer treated at the instituto nacional de cancerologia-mexico from 2004 to 2017 (*n*= 95)

Variable	β	(95% CI)
NUMB expression^a^	2.074	(0.358, 4.506)
Age	0.092	(0.015, 0.168)
HC	–0.973	(–2.541, 0.595)
NOTCH1 expression^b^	–3.428	(–5.525, 1.728)

^a^NUMB expression was defined as any positive immunostaining in the cell cytoplasm ^b^NOTCH1 expression was defined as any positive immunostaining in the cell nucleus. Age was included as a continuous variable. HC: Hormonal contraception use. β = Estimate for the association between NUMB expression and patient characteristics. 95% CI = 95% confidence interval. Bold: statistically significant.

### Effect of NOTCH1 expression on prognosis

**Figure 4:**
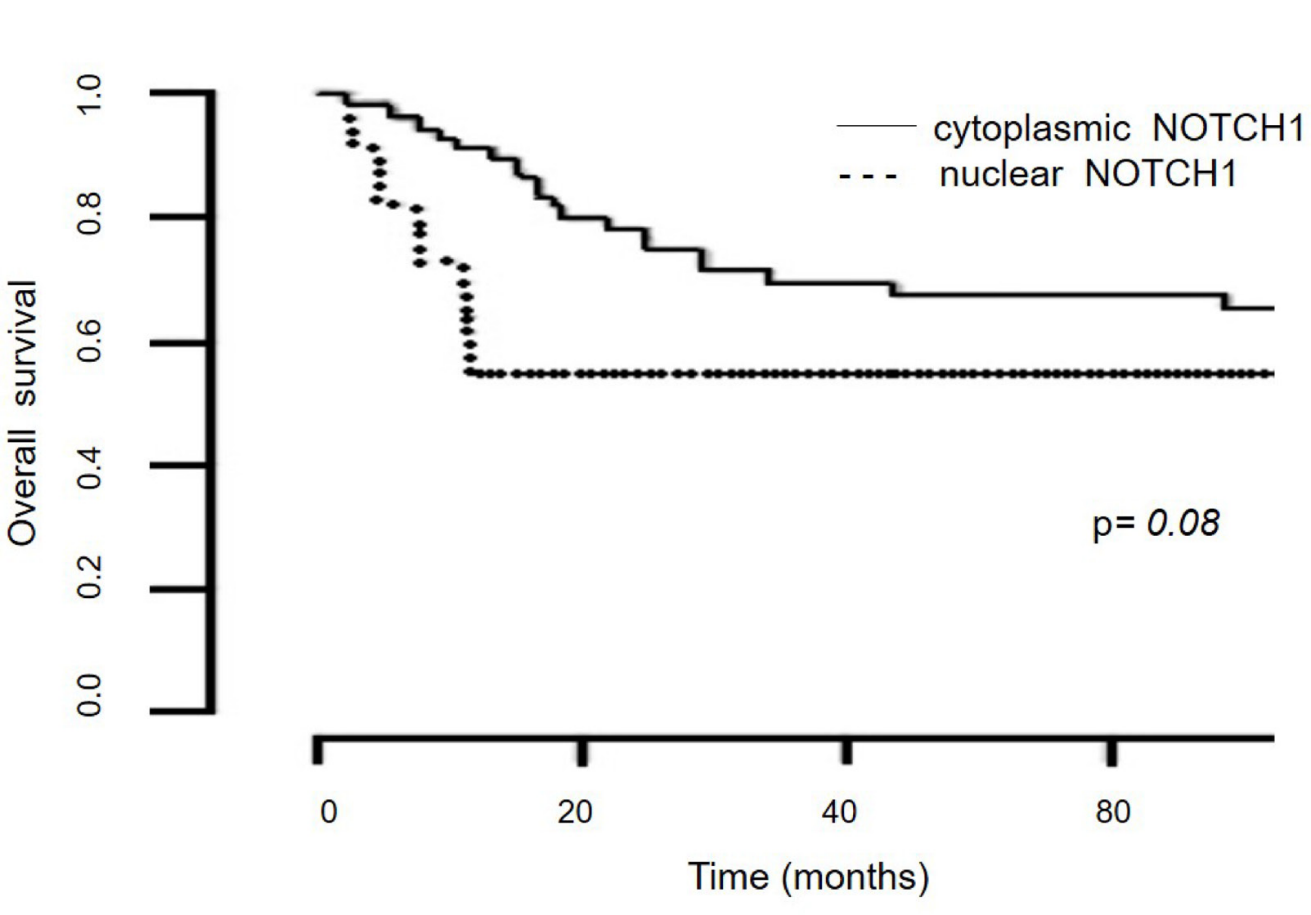
Kaplan-Meier survival analysis of the NOTCH1 localization status Overall survival according to the NOTCH1 localization status in patients with ICC treated at the Instituto Nacional de Cancerología-Mexico from 2004 to 2017 (*n* = 95).

To determine the potential effect of NOTCH1 on cervical cancer prognosis, we explored the association of NOTCH1 expression with overall survival (OS) in patients with malignant lesions. Patients with cytoplasmic NOTCH1 expression showed a longer OS than those with nuclear NOTCH1 expression, but it was only a borderline association (*P* = 0.08) (Figure 4).

## DISCUSSION

In the present study, we showed that ICC samples exhibited lower NOTCH1 expression than CIN samples and that this differential expression is also related to higher NUMB expression. We also showed that nuclear NOTCH1 expression is negatively associated with malignancy independent of known risk factors for ICC, including age and use of hormonal contraceptives as well as NUMB expression. In contrast, the association of NUMB with malignancy was not independent; it relied on NOTCH1 expression. Based on these findings, we conclude that the loss of nuclear NOTCH1 might be a key factor involved in cervical carcinogenesis. To our knowledge, this is the first study to clarify the expression and localization of NOTCH1 and NUMB in cervical cancer.

It is well known that a persistent infection with high-risk HPV is an etiological factor for cervical cancer [26]. All our samples were positive for HPV infection according to molecular examination, and the main HPV types were 16 and 18, as expected for the Mexican population, with no differences between groups [27]. The HPV type was not related to the expression of either NOTCH1 or NUMB. Concomitant factors for HPV infection and CIN development include: age, menarche, parity, age of first intercourse, number of sexual partners, use of hormonal contraceptives, body mass index, smoking and alcohol consumption [28]. Accordingly, we found that women in the cancer group were older than those in the CIN group. This finding is expected since ICC has a peak age incidence around the fourth decade of life [29].

Alterations in NOTCH signaling have been associated with tumorigenesis, but its activity is dissimilar among cancer types [22, 30–32]. In ICC, the role of the NOTCH pathway is controversial [10, 12, 13]. Some authors have proposed that NOTCH1 expression levels are stage-specific: 1) in early lesions, NOTCH1 expression is upregulated; and 2) in malignant lesions, NOTCH1 expression is downregulated [33]. In our samples, the distribution of positive cases was not different between groups, but NOTCH1 immunostaining was weaker in malignant lesions, suggesting a reduction of the expression of NOTCH1 protein.

In order to confirm the obtained results, NOTCH1 protein expression was evaluated by Western blot in ICC available cases, demonstrating coincidence with the immunohistochemical analysis which supports the loss of NOTCH1 expression in ICC.

Moreover, NOTCH1 localization has been used as a marker of activation [34], since signaling transduction relies on NICD nuclear translocation. In our samples, the loss of nuclear immunoreactivity in ICC samples, could be related to inactivation of the canonical pathway. This could be confirmed by the analysis of the expression of NOTCH1 target genes, such as those belonging to the *Hes* or *Hey* family [7, 9].

Chen *et al.* (2009) showed that NUMB expression increased during cervical carcinogenesis [24]. Increased NUMB expression has also been reported in oral squamous cell carcinoma (OSCC) [31], hepatocellular cancer (HCC) [35] and endometrial cancer [36]. In contrast, other studies have suggested NUMB as a tumor suppressor protein [37]. NUMB downregulation has been reported in breast cancer and non-small-cell lung carcinomas (NSCLCs), probably due to extensive degradation [22, 38]. We found an increase in NUMB expression in ICC compared to CIN, and this overexpression of NUMB correlates with a decrease in NOTCH1 expression. Even more, we found that premalignant lesions tended to express NUMB in the nucleus which is upregulated in cervical cancer with a cytoplasmic localization. Besides, cytoplasmic NUMB expression was associated with a decrease in nuclear NOTCH1 expression in cervical cancer samples, suggesting the potential regulation of NUMB over NOTCH1, as previously suggested [17, 39]. In the correlation analysis, we expected that tissue areas with NUMB positive expression would correlate with negative NOTCH1 areas. In CIN samples, we did not find a correlation, but we found low positive correlation between the expressing areas of NOTCH1 and NUMB in ICC reflecting that both proteins are present in the cytoplasm in the transformed tissue. This could suggest, that a cytoplasmic NUMB isoform expressed in ICC, could avoid NICD nuclear translocation and thus, inhibit its activity [19, 40].

As previously mentioned, the differences in NUMB localization in our groups (CIN vs. ICC) might be due to different isoform expression. *NUMB* mRNA can be alternatively spliced, giving rise to at least six isoforms of the protein with differences in the size of the phosphotyrosine-binding domain (PTB) and the proline-rich region (PRR) [41]. However, analysis of the NUMB isoforms is beyond the aims of our study since there are no isoform-specific NUMB antibodies available.

The expression of another suppressor as p63 [33, 47]. It is worth to mention that the protein Atlas database report a moderate NOTCH1 immunoexpression in cervical cancer, with a heterogeneous localization in the cell [25]; this is opposite to our results since we found a diminished NOTCH1 expression in cervical cancer. We are not able to distinguish the membranal protein since our antibody is against the intracellular domain. On the other hand, our results of NUMB expression agree with those reported in the Protein Atlas since in both cases the expression is strong with a cytoplasmic localization.

NOTCH1 expression has been proposed as a poor prognostic factor for many types of cancer, such as breast, gastric and lung cancer [42–44]. In this study, we showed that nuclear NOTCH1 expression was negatively correlated with malignancy status, this means that loss of nuclear NOTCH1 expression, the presumably active protein, might favor neoplastic progression from precursor cervical lesions (CIN) to cancer (ICC). Similar findings were recently observed in a study of small cell lung cancer, in which high NOTCH1 expression was an independent favorable prognostic factor [45].

Besides, we found that patients with ICC and cytoplasmic NOTCH1 expression tended to exhibit longer OS compared with those with nuclear NOTCH1 expression. Our findings related to OS are limited by the low number of ICC samples used for this analysis. Poor survival of patients with nuclear NOTCH1 and 3 expressions has already been described in non-small cell lung cancer and cervical carcinoma [44, 46].

The data showed here, support the results of Talora *et al.* (2012) that NOTCH1 is downregulated in later stages of cervical carcinogenesis. Thus, NOTCH1 seems to have a suppressive function in ICC. Moreover, Sun *et al.* (2009) found a similar behavior for NOTCH1, demonstrating its suppressive role through the induction of We acknowledge that the present analysis has several limitations, including that this is a retrospective study based mainly in paraffin embedded tissue; therefore, we lack enough fresh biological material to perform immunofluorescence for co-localization confirmation. Moreover, since our Institution is a cancer reference center, we could collect only a relatively low number of samples in the CIN group. Still, the consistency of the results from our sensitivity analysis suggests that the sample size was not a limitation. Additionally, we obtained a limited number of ICC samples exhibiting nuclear NOTCH1 expression. Nonetheless, the present study is distinctively unique due to the analysis of NOTCH1 and NUMB expression and localization in CIN and ICC samples.

In conclusion, nuclear NOTCH1 is highly expressed in premalignant lesions, while the lack of nuclear NOTCH1 is an independent predictor of malignancy. Additionally, the association of NUMB with malignancy is dependent on NOTCH1 expression. We propose that the loss of nuclear NOTCH1 may contribute to cervical carcinogenesis. These results point to target the NOTCH pathway as a therapeutic in cervical cancer.

## MATERIALS AND METHODS

### Tissue specimens

A total of 144 biopsy tissue samples were collected from January 2004 to December 2016 in the Pathology Department of the Instituto Nacional de Cancerología in México City: 95 ICC samples (11 adenocarcinomas and 84 squamous cell carcinomas) and 49 cervical premalignant lesions (including 29 low-grade squamous intraepithelial lesions: CIN I and II; and 20 high-grade lesions: CIN III and *in situ* carcinoma). Hematoxylin and eosin staining confirmed histopathological diagnoses. Clinical and pathological parameters were collected from the medical files. This project was approved by the Institutional Review Board (INCAN/Of.CEI577/15).

### Antibodies

The antibodies used for immunohistochemical staining were polyclonal antibodies against the C-terminus of human NUMB isoforms (p65/p66 and p71/p72) (Santa Cruz Biotechnology Inc. Dallas, Texas. sc-15590) (1:30) and activated NOTCH1 (against the cleaved intracellular fragment, NICD) (Millipore, Merck, New Jersey, USA. 07-1231) (1:50). The NOTCH1 C-20 polyclonal antibody (Santa Cruz Biotechnology Inc. Dallas, Texas, sc-6014) (1:50) was used for immunostaining validation.

### Immunohistochemical assays

Immunohistochemical staining was performed in serial sections of paraffin-embedded tissues. The slides were incubated at 60° C for 1 h, dewaxed in xylene and rehydrated in alcohol. Antigen retrieval was performed by boiling the tissues for 1 min in 0.1 mol/L citrate buffer (pH = 6) at 80° C, incubating for 30 min in a water bath, and cooling down for 5 min. Subsequently, the slides were incubated for 20 min in 0.3% H_2_O_2_ blocking buffer (EnVisio System-HRP, Dako, California, USA). Slides were incubated with the corresponding antibody overnight at 4° C in a wet chamber and later washed with phosphate-buffered saline (PBS pH 7.4) prior to incubation with anti-mouse secondary antibody (DakoCytomation EnVisio System-HRP, California, USA) for 30 min. Positive staining was detected with 3,3′-diaminobenzidin, and then, the slides were counterstained with Mayer’s hematoxylin (Merck, New Jersey, USA). Finally, the slides were preserved with rapid mounting media (Merck) and covered with a glass coverslip. Normal cervical epithelium was included as a control, accordingly to the protein Atlas database (https://www.proteinatlas.org/) that showed positive immunoreaction for NOTCH1 and NUMB proteins. Primary antibodies were replaced with PBS for the negative controls.

### Evaluation of immunohistochemical staining

The immunohistochemical evaluation was performed by a senior pathologist and an experienced examiner of the Instituto Nacional de Cancerología-México in a double-blind fashion. A Nikon ECLIPSE E200 optical microscope with a 10x eyepiece and 10x and 40x objective lens was used. The staining intensity was defined as: weak (including + and ++), when the immunoreaction was visible only with a 40x objective; and intense (+++), when staining was visible even using a 10x objective. Localization was categorized as membranal, cytoplasmic, nuclear, or combinations among them. Percentage of positivity was assessed by quartiles. Technique validation was performed using an alternative NOTCH1 antibody in a representative set of samples.

### Western blotting

NOTCH1-antibody specificity was assessed by Western Blot analysis in 22 random paraffin embedded tissues. Protein extraction was performed using the Qproteome FFPE Tissue Kit (Quiagen, Hilden, Germany). Briefly, 5 serial sections from the same block were cut with a thickness of up to 15 μm and placed in a 1.5 ml collection tube. For deparaffinization, 1 ml xylene was added into the tube and vortexed vigorously for 10 s and incubated for 10 min at room temperature (15–25° C), 100 μl of extraction buffer was added and heated. After centrifugation, the supernatant was transfered into a new tube. Total protein quantification was determined with bicinchoninic acid. After protein electrophoresis, proteins were transferred to a nitrocellulose membrane and tested with NOTCH1 (Millipore, Merck, New Jersey, USA, 07-1231) and GAPDH antibodies (Santa Cruz Biotechnology, Dallas, Texas). Clarity kit (Bio Rad, California, USA) was used for chemiluminescent protein detection. Densitometric analysis was performed using ImageJ software (Image Processing and analysis in Java).

### HPV detection

HPV typing was performed as previously described [27]. Primers from the L1 region were used (MY09/MY11/HMB01 and L1C1/L1C2.1/L1C2.2). DNA from HeLa and CaSki cells was used as a positive control. Products were analyzed by electrophoresis on 2% agarose gels stained with ethidium bromide. PCR products were directly sequenced with the BigDye Terminator v3-1 Cycle Sequencing Kit (Applied Biosystems). HPV sequences were aligned using BLAST software (http://www.ncbi.nlm.nih.gov, NCBI GenBank).

### Statistical analysis

We evaluated the different distributions of NOTCH1 and NUMB expression, intensity and localization among groups (CIN vs. ICC) using the chi-squared test. We also explored correlations between NOTCH1 and NUMB expression in histological sections using Pearson coefficients. Multivariate analysis was conducted to determine the association between NOTCH1/NUMB expression and cancer status. For the association between NOTCH1 and NUMB in malignancy, we included the following confounders: age (continuous) and hormone contraception (categorical). We used three sets of models: unadjusted; adjusted for age; and adjusted for age and hormone contraception. We used the Akaike information criterion (AIC) to evaluate goodness-of-fit. The effect of nuclear NOTCH1 expression on OS was observed using the Kaplan and Meier method, and the log-rank test was used to compare groups. All analyses were performed using R software (R Project for Statistical Computing, Wien).

## SUPPLEMENTARY MATERIALS FIGURES AND TABLES



## Abbreviations

ICC: Invasive cervical cancer
CIN: Cervical intraepithelial neoplasia
HPV: Human papilloma virus
HR: High risk
NICD: Notch intracellular domain
OS: Overall survival
OSCC: Oral squamous cell carcinoma
HCC: Hepatocellular carcinoma
PTB: Phosphotyrosine-binding domain
PRR: Proline-rich region
NSCLC: Non-small cell lung carcinoma
