# Supplementary material for: Loss of nuclear NOTCH1, but not its negative regulator NUMB, is an independent predictor of cervical malignancy
Source: Oncotarget. 2018 Apr 10;9(27):18916–28. doi: 10.18632/oncotarget.24828 (PMC5922366; doi:10.18632/oncotarget.24828)
Supplement: Supplementary file 1 [file oncotarget-09-18916-s001.pdf]

Loss of nuclear NOTCH1, but not its negative regulator NUMB, is an independent predictor of cervical malignancy

SUPPLEMENTARY MATERIALS

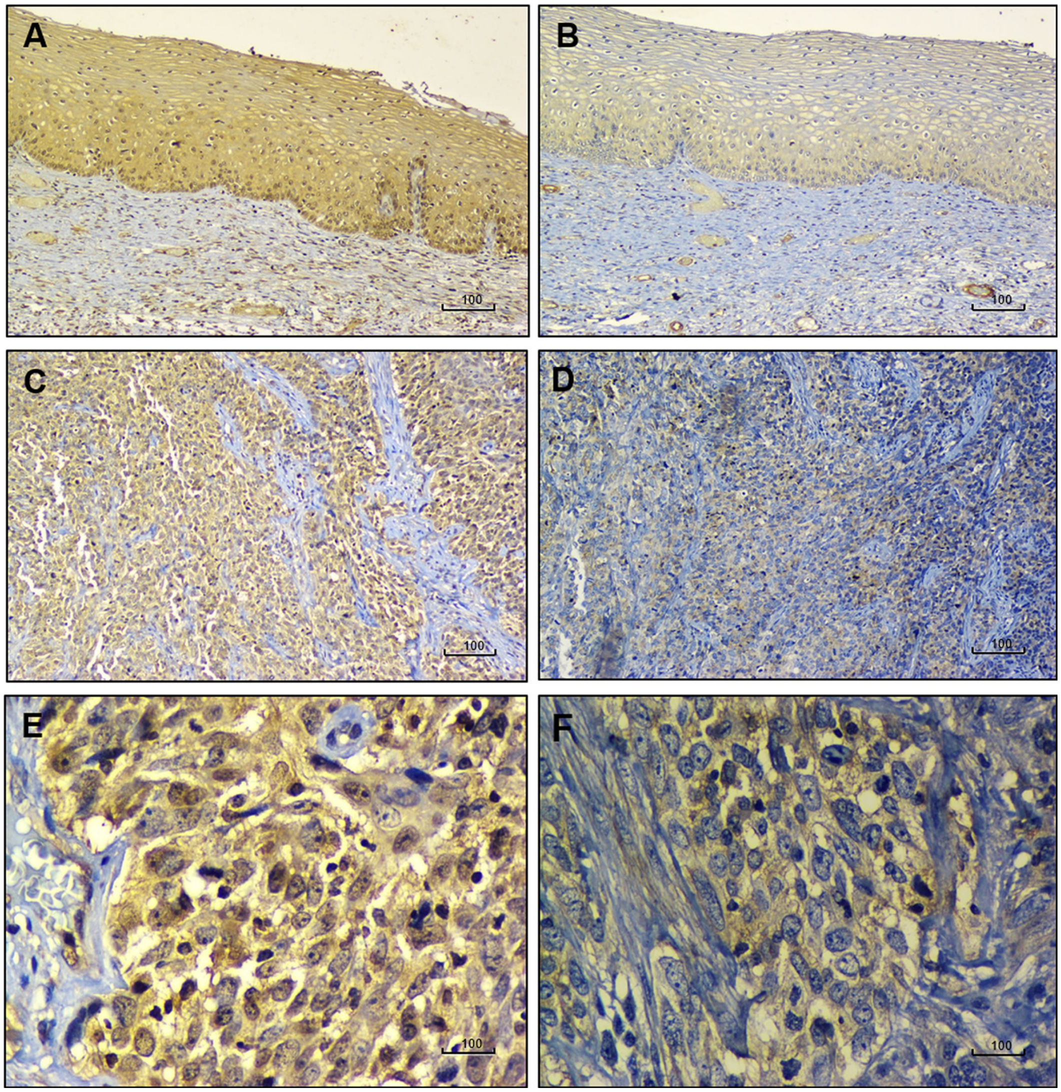

Supplementary Figure 1: Comparison between NOTCH1 antibodies immunostaining. NOTCH1 positive immunostaining with C-20 antibody of Santa Cruz Biotechnology (**A**, **C**, and **E**) and Millipore NICD antibody (**B**, **D** and **F**) in three samples: A/B, C/D and E/F.

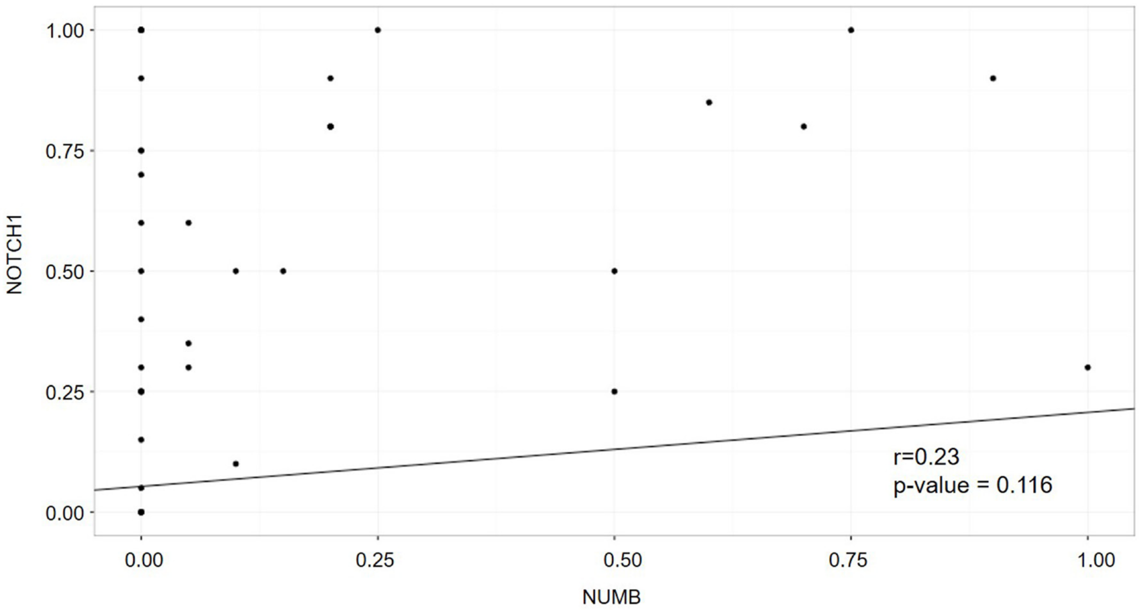

Supplementary Figure 2: Correlation between NOTCH1 and NUMB expression in CIN. Percentage of positive immunostained areas of NOTCH1 and NUMB showed no correlation in precursor cervical lesions.

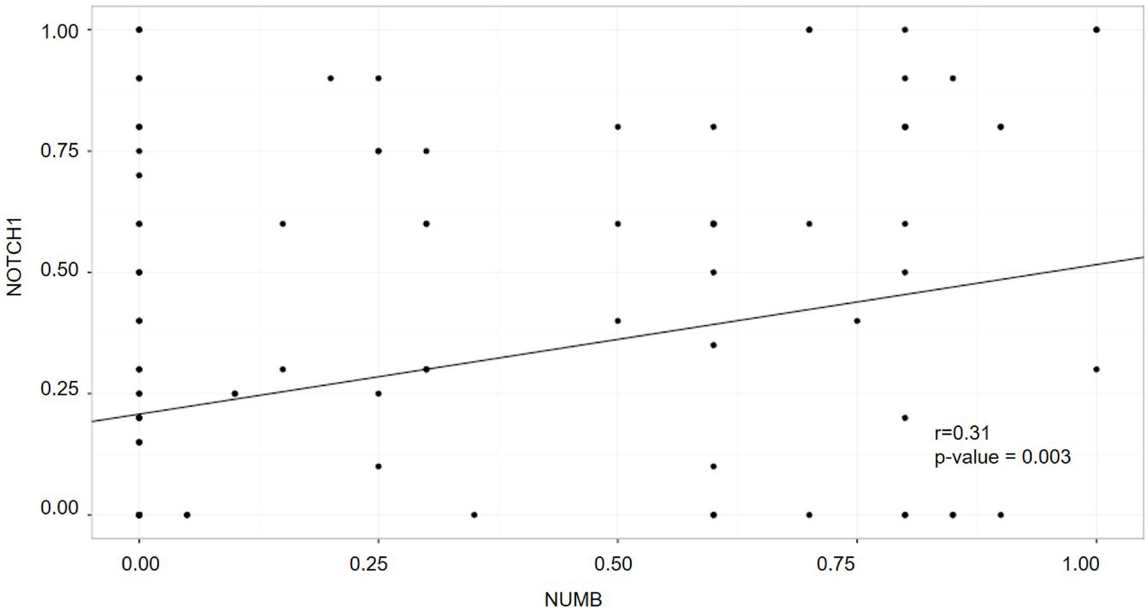

Supplementary Figure 3: Correlation between NOTCH1 and NUMB expression in ICC. Percentage of positive immunostained areas of NOTCH1 and NUMB showed a marginally positive ($r = 0.31$) and statistically significant ($p = 0.003$) correlation in cervical tumor samples.

Supplementary Table 1: Sensitivity analysis for the association between NOTCH1 expression and malignancy in patients with cervical cancer treated at the Instituto Nacional de Cancerología-México from 2004 to 2017 (*n* = 95)

	β	95% CI	*P*-value	β	95% CI	*P*-value	β	95% CI	*P*-value	β	95% CI	*P*-value
NUMB[a]	–2.836	(–3.694, –1.978)	**0.001**	–3.122	(–4.180, –2.063)	**0.001**	–3.790	(–5.459, 2.120)	**0.001**	–3.428	(–5.127, –1.728)	**0.001**
Age				0.099	(0.055, 0.142)	**0.001**	0.088	(0.013, 0.162	**0.022**	0.092	(0.015, 0.168)	**0.018**
HC							–1.300	(0.209, –2.809)	0.091	0.973	(–0.595, 2.541)	0.223
NOTCH[b]										2.074	(-0358, 4.506)	0.094
AIC		136.47			109.5			56.374			54.72	

[a]NUMB expression was described as weak to intense cytoplasmic immunostaining. [b]NOTCH1 expression was defined as weak to intense nuclear immunostaining. HC: Hormonal contraceptive use. β = estimate for the association between NUMB expression and patient characteristics. 95% CI = confidence interval. AIC: Akaike information criteria. Bold: statistically significant.

Supplementary Table 2: Sensitivity analysis for the association between NUMB expression and malignancy in patients with cervical cancer treated at the Instituto Nacional de Cancerologia-Mexico from 2004 to 2017 (n = 95)

	β	95% CI	*P*-value	β	95% CI	*P*-value	β	95% CI	*P*-value	β	95% CI	*P*-value
NUMB[a]	3.286	(5.310, 1.262)	0.001	3.487	(5.548, 1.427)	**0.001**	2.946	(0.809, 5.082)	**0.007**	2.074	(–0.358, 4.506)	0.094
Age				0.091	(0.129, 0.054)	**0.001**	0.072	(–0.024, 0.076)	**0.005**	0.092	(0.015, 0.168)	0.018
HC							–0.844	(–2.027, 0.339)	0.162	–0.973	(–2.541, 0.595)	0.223
NOTCH[b]										–3.428	(–5.127, –1.728)	**0.001**
AIC		162.63			128.77			75.546			54.722	

[a]NUMB expression was described as weak to intense cytoplasmic immunostaining. [b]NOTCH1 expression was defined as weak to intense nuclear immunostaining. HC: Hormonal contraceptive use. β = estimate for the association between NUMB expression and patient characteristics. 95% CI = confidence interval. AIC: Akaike information criteria. Bold: statistically significant.

Supplementary Table 3: Comparison of immunostainig detection of two NOTCH1 antibodies in ICC samples (*n* = 10)

Case	NOTCH1 (Millipore, Merck[a])	NOTCH1 (Santa Cruz Biotechnology[b])
1	Positive	Positive
2	Positive	Positive
3	Positive	Positive
4	Positive	Positive
5	Negative	Positive
7	Positive	Positive
8	Negative	Negative
9	Positive	Positive
10	Positive	Positive

[a]Millipore Merck antibody is against activated NOTCH1 (against the cleaved intracellular fragment, NICD) (Millipore, Merck, New Jersey, USA. 07-1231). [b]Santa Cruz Biotechnology antibody detects full-length NOTCH1 as well as cleaved NOTCH1 (Santa Cruz Biotechnology Inc. Dallas, Texas, sc-6014).
